# Methodological considerations for near-infrared spectroscopy assessments in rock climbers: impact of forearm morphology and optode placement

**DOI:** 10.3389/fspor.2026.1845130

**Published:** 2026-06-03

**Authors:** Simon Vandenhaute, Ota Podlipný, Eliška Čikotová, Tobia Forrer, Jiří Baláš

**Affiliations:** 1 Department of Athletics and Outdoor Sports, Faculty of Physical Education and Sport, Charles University, Prague, Czechia; 2 Department of Physiotherapy, Faculty of Physical Education and Sport, Charles University, Prague, Czechia; 3 Department of Neuroscience, Biomedicine and Movement Sciences, University of Verona, Verona, Italy; 4 CeRiSM – Research Center for Sport, Mountain and Health, University of Verona, Rovereto, Italy

**Keywords:** haemodynamics, intermittent endurance, muscle oxygenation, muscle thickness, vascular occlusion, flexor digitorum profundus, flexor digitorum superficialis

## Abstract

**Introduction:**

Near-infrared spectroscopy (NIRS) has been widely used in rock-climbing research to assess muscle oxygenation (StO_2_) in the flexor digitorum profundus (FDP). However, the deep anatomical location of the FDP, combined with the maximum penetration depth of ∼20 mm for most NIRS devices, raises uncertainty regarding whether the currently used optode placement is adequate for studying the FDP. This study aimed to characterize forearm morphology in climbers and examine the effect of NIRS optode placement on haemodynamic outcomes.

**Methods:**

This study comprised two parts. In Part 1 (*n* = 28), adipose tissue thickness (ATT), flexor digitorum superficialis (FDS) thickness, and FDP thickness were measured at three forearm sites (proximal, reference, and distal; 20 mm spacing) in supination and pronation using ultrasound. The reference site was defined as 33% of the distance between the medial epicondyle and lunate. Part 2 (*n* = 22) consisted of a vascular occlusion and incremental finger flexor endurance test with NIRS optodes positioned either perpendicular or parallel to the FDP fibres. The outcomes included half-time recovery of reoxygenation (StO₂ HTR) from the vascular occlusion test, reoxygenation between contractions during the first (*Δ*StO_2 relief—first_) and final stages (*Δ*StO_2 relief—last_), and mean oxygenation (StO_2 mean_) during the incremental test.

**Results:**

The FDS–FDP border was located 17.6 ± 4.0 mm deep, indicating that the FDS dominated the near-infrared signal. During the incremental test, *Δ*StO_2 relief—last_ was 2.7 ± 2.9% lower (*p* < 0.001, *g* = 0.87) and StO_2 mean_ was 5.5 ± 3.4% higher (*p* < 0.001, *g* = 1.56) with perpendicular optode orientation than with parallel optode orientation. StO_2_ HTR and *Δ*StO_2 relief—first_ were not different between optode orientations (*p* = .145, *g* = 0.33; *p* = 0.799, *r_rb_* = 0.06).

**Discussion:**

In conclusion, only a small portion of the FDP lies within the NIRS penetration depth. StO_2_ reoxygenation between contractions was more pronounced when the optodes were aligned parallel to the muscle fibres, although this was observed only at moderate intensities. Based on these findings, we recommend positioning the NIRS optodes over the reference site, aligning them parallel to the muscle fibres, and using the term “flexores digitorum” when referring to the muscles under investigation.

## 1 Introduction

Rock climbing requires repeated isometric contractions of the finger flexor muscles, with only brief periods of relaxation. During sustained gripping efforts, intramuscular pressure increases, occluding blood flow and limiting oxygen delivery to the working muscles. Therefore, the ability to rapidly restore oxygenation during short relaxation phases is considered a key determinant in climbing performance (1–4).

Near-infrared spectroscopy (NIRS) has become an increasingly popular method for non-invasively investigating skeletal muscle oxygenation and haemodynamics in sports science (5). By measuring light absorption changes in oxygenated and deoxygenated haemoglobin and myoglobin, NIRS provides insights into local muscle haemodynamics. In climbing research, both vascular occlusion protocols of the brachial artery and intermittent endurance tests of the finger flexors have been used to assess forearm oxygenation (6–8). The rate of reoxygenation after vascular occlusion, along with the extent of reoxygenation during intermittent contraction tests, has shown good reliability and is associated with climbing ability (1, 4, 9, 10).

Although both the flexor carpi radialis (FCR) (11, 12) and flexor digitorum superficialis (FDS) (3) have been studied in climbers, the flexor digitorum profundus (FDP) has received the greatest attention (13–17).

Despite the widespread use of NIRS to study the FDP, no standardized protocol for optode placement has been established. In most studies assessing FDP haemodynamics with NIRS, the measurement site was chosen at 33% of the distance from the medial epicondyle of the humerus to the lunate bone, starting from the medial epicondyle (1, 10). Alternatively, some studies have used palpation along this line to identify the site where the FDP appears most prominent before positioning the NIRS probe (4, 9).

The relevance of optode positioning must be considered in relation to the maximal penetration depth of NIRS, which is approximately half of the maximal inter-optode distance (18). Thus, with a typical maximum distance between the optodes of 40 mm, measurements will reach depths of approximately 20 mm. Given that the FDP is located deep within the anterior compartment of the forearm, this limited penetration depth raises uncertainty about whether current methodologies can accurately capture its haemodynamic responses.

Another methodological factor that has received little attention is the orientation of NIRS optodes relative to the underlying muscle fibres. No studies have examined the orientation of optodes with respect to the direction of muscle fibres. However, muscle tissue exhibits anisotropic optical properties, which means that the propagation of near-infrared (NIR) light can vary depending on the orientation of the fibres (19). For instance, in the human biceps brachii muscle, photons are more likely to migrate parallel to rather than perpendicular to the muscle fibres (20). Therefore, optode orientation may substantially influence NIRS outcomes, and the observed differences could reflect optical artefacts rather than true physiological differences.

Considering these methodological factors, gaining a better understanding of forearm morphology in climbers and the effects of optode placement is required to improve the interpretation and comparability of NIRS measurements in climbing research.

The aims of this study were as follows: (1) to characterize forearm morphology in climbers across different sites by quantifying the tissue thickness of the FDP and FDS; and (2) to examine the effect of NIRS optode orientation relative to the direction of muscle fibres on muscle oxygenation and haemodynamic responses during rock-climbing-related tasks.

## 2 Methods

### 2.1 Study design

To achieve the research objectives, the study was divided into two parts: Characterization of forearm muscle morphology using ultrasound (US) (Part 1) and examination of the haemodynamic responses of the finger flexors using NIRS (Part 2).

In Part 1, a cross-sectional observational design was employed to assess the muscle thickness of the FDS and FDP, as well as the adipose tissue thickness (ATT) of the dominant forearm. Measurements were obtained at three standardized sites on the forearm and in two different positions (supination and pronation). Moreover, reliability measures were taken for a part of the research sample.

Part 2 employed a within-subject, cross-over design to investigate the effects of NIRS-optode orientation (parallel vs. perpendicular to the muscle fibres of FDP) on haemodynamic measures during a single laboratory visit. For each optode orientation, the participants performed one vascular occlusion of the dominant upper arm and one sub-maximal incremental exercise test for the finger flexors. The order of optode orientation was randomized to mitigate potential order effects. Participants were instructed to refrain from strenuous exercise for 24 h, caffeine for 12 h, and food for 3 h before testing commenced.

### 2.2 Participants

Twenty-eight climbers [nine female and 19 male; age, 27.7 ± 8.5 years; body mass, 71.1 ± 14.0 kg; height, 176.7 ± 8.8 cm; lead performance on the International Rock-Climbing Research Association (IRCRA) grading scale (21), 17 ± 4; mean ± standard deviation (SD)] volunteered to participate in Part 1. Twenty-two participants (seven women and 15 men; age, 27.5 ± 8.7 years; body mass, 67.0 ± 9.4 kg; height, 175.0 ± 8.1 cm; lead performance on the IRCRA grading scale, 18 ± 4; mean ± SD) were recruited for Part 2. Most participants took part in both the ultrasound and NIRS experiments; however, additional participants were recruited for each study component to complete the respective samples. All participants regularly exercised their upper body and finger flexors. The study was approved by the local Ethics Committee (EK 140/2025, FTVS) and was conducted in accordance with the Declaration of Helsinki. All participants received written and verbal information about the study procedures and provided written informed consent prior to their participation.

### 2.3 Ultrasound

US images and measurements were obtained and analysed by a trained physiotherapist using a Samsung HS30 (Samsung Electronics Co., Ltd., Suwon, South Korea) with a linear L5-12/50 probe (5–12 MHz) operating in B-mode.

**Figure 1:**
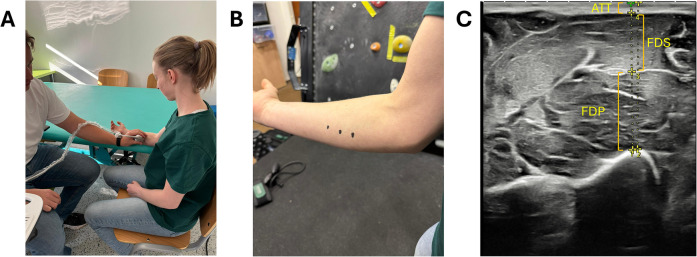
**(A)** The participant's position during the ultrasound measurements. **(B)** The marks with 20 mm intervals along the line connecting the medial epicondyle of the humerus and the lunate bone. The middle (reference) mark was placed at 33% of the distance, measured from the medial epicondyle. **(C)** An example of an ultrasound image and the measured tissue thickness. ATT, adipose tissue thickness; FDS, flexor digitorum superficialis; FDP, flexor digitorum profundus.

Participants were seated with the forearm resting on a table, the wrist fully supinated, the elbow in approximately 120° extension, and the shoulder abducted approximately at 30° (Figure 1A). The distance between the medial epicondyle of the humerus and the lunate bone was measured using a flexible tape measure. Following previously published protocols (1), a mark was placed on the reference site, specifically at 33% of the distance measured from the medial epicondyle. Two additional marks were placed on the same line: one 20 mm proximal and the other 20 mm distal to the reference mark (Figure 1B). Prior to testing, the forearm circumference was measured at the reference site using a flexible tape measure.

For each site, the middle of the ultrasound probe was aligned with the mark and positioned perpendicular to the line connecting the medial epicondyle of the humerus and the lunate bone. At the reference site, an additional scan was obtained with the forearm fully pronated, as this is a more climbing-specific position. In total, four measurement conditions were assessed: proximal site—supinated, reference site—supinated, distal site—supinated, and reference site—pronated.

Using the built-in measurement software of the US device, tissue thickness was measured along a line perpendicular to the skin surface, extending to the nearest visible point on the ulna (Figure 1C). For each condition, the following thicknesses were recorded: ATT, FDS, and FDP. Furthermore, US images at each site were assessed for the presence of flexor carpi ulnaris (FCU), FCR, and palmaris longus (PL). Different muscles were identified by a trained physiotherapist based on the US images. When in doubt, participants were instructed to perform selective contractions to facilitate identification. The specific finger associated with the muscle portion located beneath the probe was not determined, as the exercise protocol, like most climbing tests, involved the simultaneous engagement of all fingers.

In addition, test–retest reliability was recorded for all four measurement conditions in a subset of eight participants.

### 2.4 Near-infrared spectroscopy

Muscle haemodynamic measures were assessed using a continuous-wave NIRS device (Portalite, Artinis Medical Systems BV, The Netherlands). The reference site was marked in the same way as described in Part 1. The optodes were positioned over the mark either parallel or perpendicular to the line connecting the medial epicondyle and lunate. The perpendicular and parallel orientations were pre-programmed and selected independently of the morphological analysis. They were chosen to represent the two extremes of optode orientation relative to the muscle fibre direction, thereby maximizing the potential differences between conditions.

When necessary, the optode position was adjusted by up to 5.0 mm to avoid the visible superficial veins. The device was fixed with a self-adhesive tape to reduce motion artefacts and covered with a black sleeve to minimize interference from ambient light. The order of the optode orientations was randomized.

Raw NIRS signals were acquired at a sampling frequency of 10 Hz and recorded using the manufacturer's software (OxySoft, Artinis Medical Systems BV, The Netherlands). The NIRS device has inter-optode distances of 30, 35, and 40 mm, resulting in a maximum penetration depth of approximately 20 mm, and emits light at wavelengths of 760 and 850 nm. A differential pathlength factor of 4 was applied for all measurements, as this is the average of previously reported differential pathlength factors in the adult forearm (22, 23).

Before each incremental test, participants warmed up and performed a maximum voluntary contraction (MVC) of the finger flexors on a 23 mm edge using a Climbro hangboard (Climbro Ltd., Sofia, Bulgaria), following the protocol outlined by Michailov et al. (24).

Muscle oxygenation (StO₂, %) was calculated using the built-in algorithm of Oxysoft. In the analysis, only StO₂ calculated via spatially resolved spectroscopy using all three optode pairs was included. StO₂ was determined as follows:

$${\mathrm{StO}}_{\text{2}}\;\left(\%\right)=\frac{{\text{O}}_{2}\text{Hb}}{{\text{O}}_{\text{2}}\text{Hb + HHb}}\times 100$$

#### 2.4.1 Vascular occlusion test

The participants were positioned supine with the dominant arm abducted to 30° and elevated on a ramp so that the forearm was at heart level. A blood pressure cuff (Occlude Athlete, Occlude, Aarhus, Denmark) was placed over the upper arm as proximally as possible.

Following a 5 min baseline recording, the cuff was manually inflated to 260 mmHg to induce vascular occlusion. The occlusion lasted 3–5 min, depending on when a plateau in StO₂, defined as a constant value for 10 s, was visually observed.

**Figure 2:**
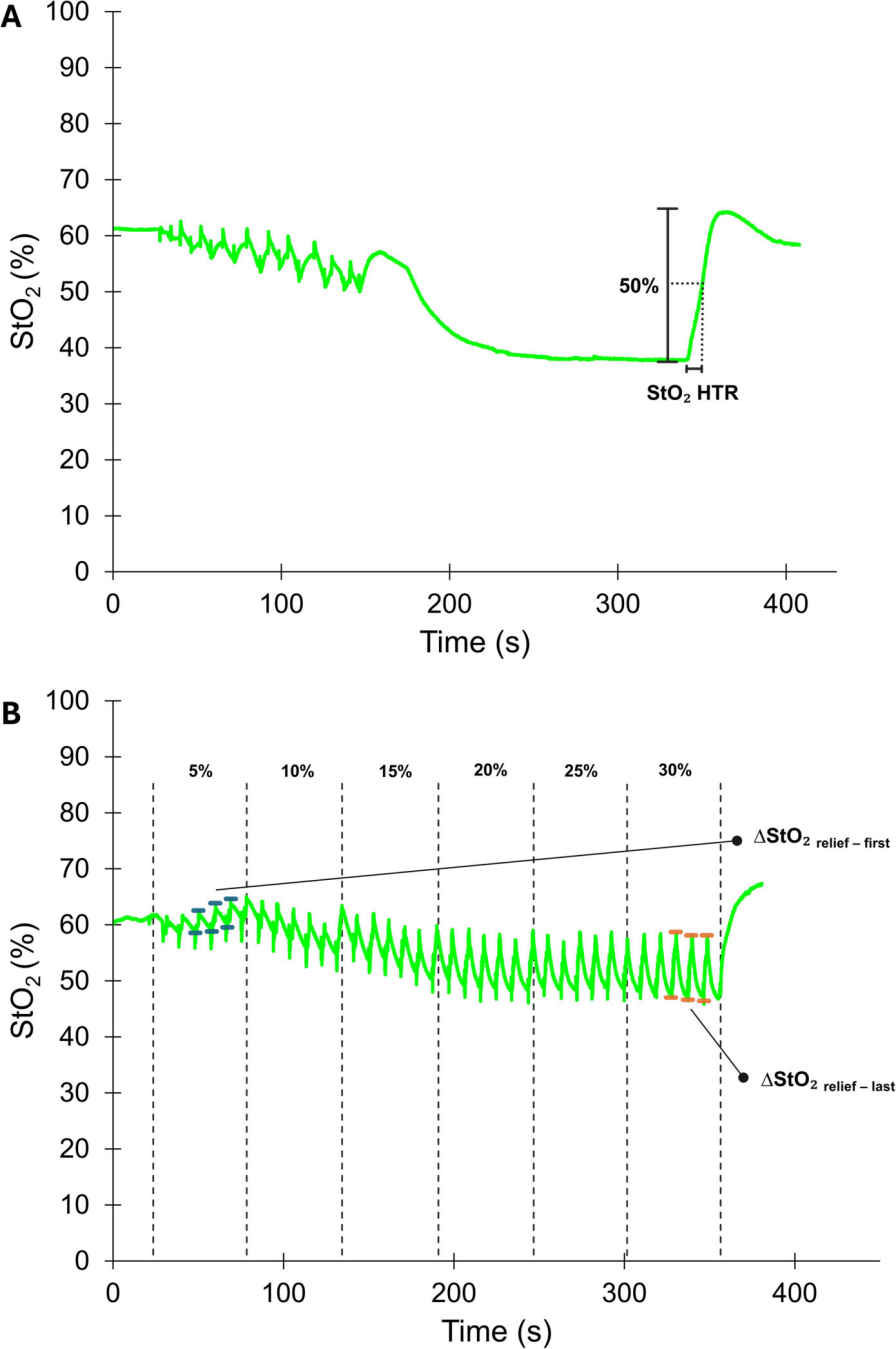
**(A)** A typical muscle oxygen saturation (StO₂) trace from a vascular occlusion test with a bar showing the half-time recovery of tissue saturation (StO₂ HTR). **(B)** An example of the StO₂ trace from an incremental test. The three relief periods for the calculation of *Δ*StO₂ recovery during the first (*Δ*StO₂ _relief—first_) and last stage *Δ*StO₂ of the test (*Δ*StO₂ _relief—last_) are marked. The different stages are delineated by dashed lines, with the percentages referring to the intensity relative to the maximum voluntary contraction.

The time to half recovery of StO₂ (StO₂ HTR, s) was defined as the time between the point of maximal deoxygenation at the end of the occlusion and the point at which 50% of the peak reoxygenation level attained during the post-occlusion hyperaemia was reached (Figure 2).

#### 2.4.2 Incremental exercise test

After a 5 min recovery period, the participants performed an incremental intermittent exercise protocol for the finger flexors using the Climbro hangboard. Before starting the protocol, the participants stood upright with their arms hanging freely beside their bodies until a stable baseline StO₂ lasting at least 30 s was visually confirmed. The test consisted of six intensity steps, starting at 5% MVC and increasing by 5% MVC at each step up to 30% MVC. Each step comprised six repetitions of a 7 s isometric contraction interspersed with 3 s rest periods. The same 3 s rest period was used between the different intensity steps. To minimize carry-over and fatigue effects, the exercise protocol was submaximal, and a wash-out period of at least 15 min was applied between the two conditions.

*Δ*StO₂ recovery during the first stage of the test (*Δ*StO₂ _relief—first_, %) and *Δ*StO₂ recovery during the last stage of the test (*Δ*StO₂ _relief—last_, %) were calculated as the mean of the StO₂ recovery of the middle three relief periods for the first and last stages, respectively (9) (Figure 2). Furthermore, the mean StO₂ (StO₂ _mean_, %) during the incremental test was calculated.

In summary, each participant completed one vascular occlusion test and one incremental exercise test with the optodes oriented perpendicular to the muscle fibres, as well as another vascular occlusion test and incremental exercise test with the optodes positioned parallel to the muscle fibres.

### 2.5 Statistical analysis

#### 2.5.1 Part 1

Linear mixed-effects models were used to examine differences in tissue thickness between the measurement sites and positions. Measurement site (proximal, reference, and distal) and sex were defined as fixed effects, and a random intercept for each subject was included to account for between-subject variability. This linear mixed-effects model was repeated with position (supinated or pronated) and sex as the fixed effects. Using the Shapiro–Wilk test, residuals were found to be normally distributed for FDS and FDP thickness but not for ATT. However, a visual investigation of the Q–Q plot of the ATT residuals showed only minor deviations from normality. *Post-hoc* pairwise comparisons were calculated using Bonferroni correction. Furthermore, the mean and SD of the tissue thicknesses were calculated.

An intraclass correlation [ICC(2,1)] was employed to analyse the test–retest reliability of the ultrasound measurements for a single rater and was calculated as follows:

$$\mathrm{ICC}=\;\frac{MS_{R}-MS_{E}}{MS_{R}+\frac{1}{n}(MS_{C}-MS_{E})}$$

where MS_R_ is the mean square for rows (subjects), MS_E_ is the mean square for error (residuals), MS_C_ is the mean squares for columns (raters), and *n* is the number of subjects. Mean squares were obtained from a two-way ANOVA. The standard error of measurement (SEM) was calculated using the following formula:

$$\mathrm{SEM}=\;\sqrt{{\mathrm{MS}}_{E}}$$

Finally, the forearm circumference was correlated with tissue thickness using Pearson's *r* correlation coefficients.

#### 2.5.2 Part 2

The paired differences of the haemodynamic outcome measures were tested for normality using the Shapiro–Wilk test. Paired *t*-tests were used to examine the differences between the perpendicular and parallel conditions. If the paired differences did not follow a normal distribution, the Wilcoxon signed-rank was used for analysis, and effect sizes were reported as rank-biserial correlations (*r_rb_*). Otherwise, all effect sizes are presented as partial eta squared (*η*_p_^2^) for the main effects of the linear mixed-effects models and Hedges' *g* for the pairwise comparisons and *t*-tests.

Statistical significance was set at *α* = 0.05 for all tests in both parts. *η*_p_^2^ values of 0.01, 0.06, and 0.14 and Hedges' *g* values of 0.2, 0.5, and 0.8 were interpreted as small, medium, and large, respectively. All statistical analyses were performed using R (version 4.5.2) (25).

## 3 Results

### 3.1 Part 1

The reference site was effective in avoiding FCR and FCU in all but one participant. At the proximal site, the FCR overlapped the FDS in 17.9% of cases, whereas at the distal site, this overlap occurred in only 7.1% of cases. The muscle belly of the PL could be identified and overlaid on the FDS in 32%, 43%, and 18% of individuals on the reference site, proximal site, and distal site, respectively.

**Figure 3:**
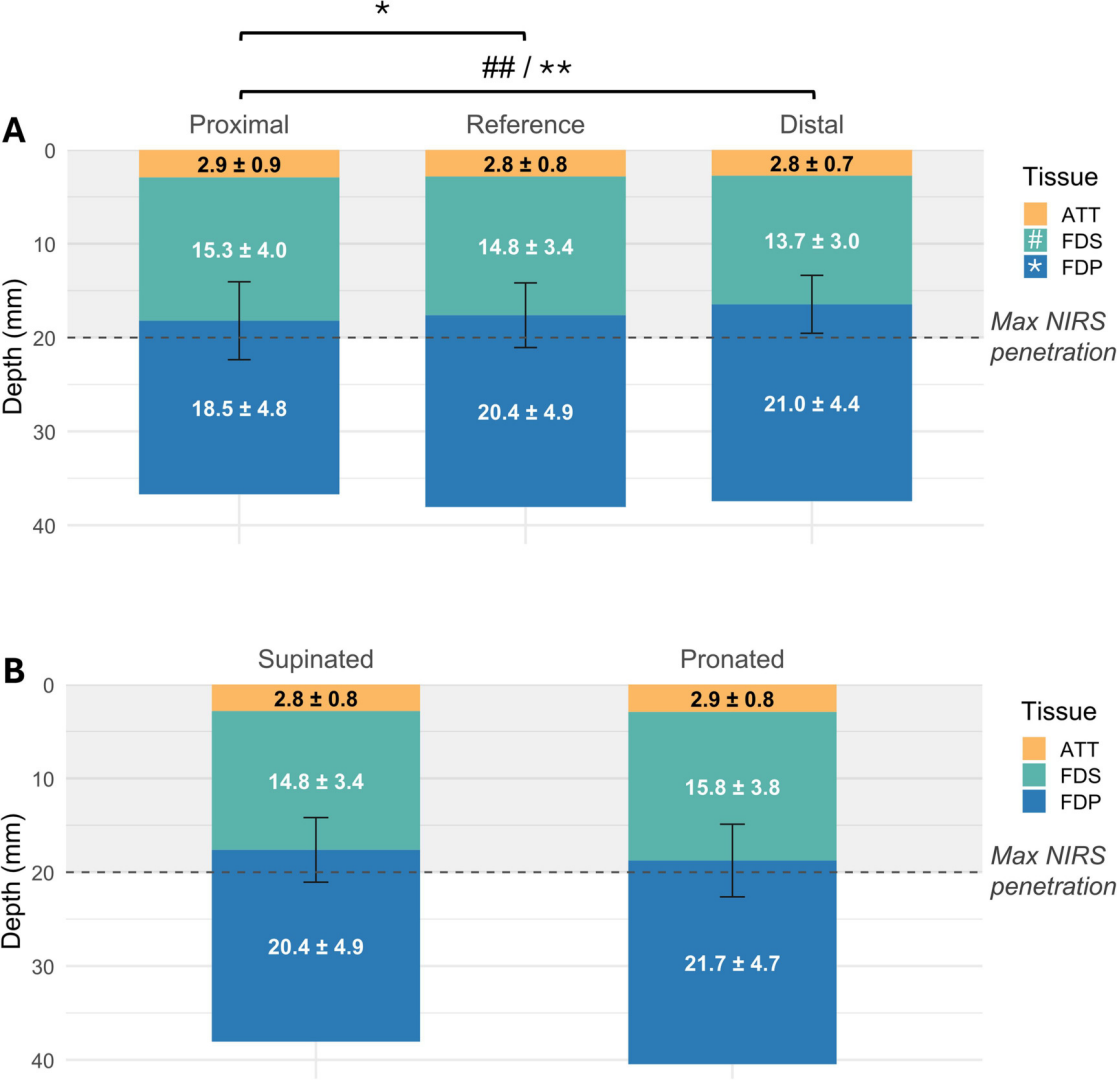
Mean ± standard deviation of tissue thickness per measurement condition. The dotted black line represents the maximal penetration depth of most commercially available near-infrared spectroscopy devices, which have a maximal inter-optode distance of 40 mm. The error bars represent the pooled standard deviation of the ATT and FDS thicknesses. **(A)** Grouped by measurement site. **(B)** Grouped by measurement position. ATT, adipose tissue thickness; FDS, flexor digitorum superficialis; FDP, flexor digitorum profundus. * Significant differences in FDP thickness (*p* < 0.05). ** Significant differences in FDP thickness (*p* < 0.01). ## Significant difference in FDS thickness (*p* < 0.01).

The mean tissue thicknesses grouped by measurement site and position are shown in Figure 3. A significant effect of the measurement site was observed on the FDS and FDP thickness. *Post-hoc* comparisons revealed that the FDS thickness was 11.5% lower at the distal site than at the proximal site (*p* = 0.007, *g* = 0.86). The FDP thickness was 11.8% lower at the proximal site than at the distal site (*p* = 0.001, *g* = 1.00) and 6.4% lower than at the reference site (*p* = 0.016, *g* = 0.78). No significant differences were detected between the supinated and pronated positions for any of the tissues (ATT, *p* = 0.228; FDS, *p* = 0.152; FDP, *p* = 0.223).

Under all measurement conditions, men presented an average FDS thickness that was 2.8 ± 1.2 mm greater (mean ± SD, *η_p_^2^* = 0.18, *p* = 0.026) and a FDP thickness that was 6.6 ± 1.2 mm thicker (mean ± SD, *η_p_^2^* = 0.55, *p* < 0.001). In contrast, no differences were found for the ATT (*η*_p_^2^ = 0.07, *p* = 0.188). Furthermore, no interaction effects between sex and the measurement site or position were detected. The means and standard deviations for tissue thickness categorized by sex are provided in the Supplementary Materials (Supplementary Table S1).

Forearm circumference showed a strong correlation with FDP thickness (*r* = 0.76; *p* < 0.001) and a weak correlation with FDS thickness (*r* = 0.44; *p* = 0.020) at the reference site.

The reliability measures are presented in Table 1. FDS thickness and ATT showed good to excellent test–retest reliability, with ICC (2,1) ranging from 0.62 to 0.93 and SEM from 0.2 to 2.0 mm. FDP thickness showed poor reliability, with ICC (2,1) ranging from 0.2 to 0.62 and SEM from 2.1 to 4.0 mm.

**Table 1:** Test–retest reliability of tissue thickness measurements at each site.

Tissue	Difference (mm)	ICC	95% CI	SEM (mm)
Proximal				
ATT	−0.2 ± 0.5	0.90	0.63–0.98	0.4
FDS	0.7 ± 2.6	0.62	−0.04–0.91	1.8
FDP	−2.6 ± 3.3	0.41	−0.16–0.83	2.3
Reference—supinated				
ATT	−0.2 ± 0.6	0.90	0.62–0.98	0.4
FDS	0.7 ± 1.5	0.78	0.30–0.95	1.1
FDP	−2.1 ± 5.0	0.39	−0.30–0.83	3.5
Distal				
ATT	−0.2 ± 0.3	0.93	0.69–0.99	0.2
FDS	0.9 ± 1.7	0.64	0.04–0.91	1.2
FDP	−1.3 ± 3.0	0.62	0.00–0.91	2.1
Reference—pronated				
ATT	−0.4 ± 0.5	0.86	0.37–0.97	0.4
FDS	0.3 ± 2.8	0.72	0.16–0.94	2.0
FDP	−2.2 ± 5.7	0.20	−0.50–0.76	4.0

ICC, intraclass correlation; SEM, standard error of measurement; ATT, adipose tissue thickness; FDS, flexor digitorum superficialis; FDP, flexor digitorum profundus. Differences were calculated as test 1−test 2 (mean ± SD).

### 3.2 Part 2

**Figure 4:**
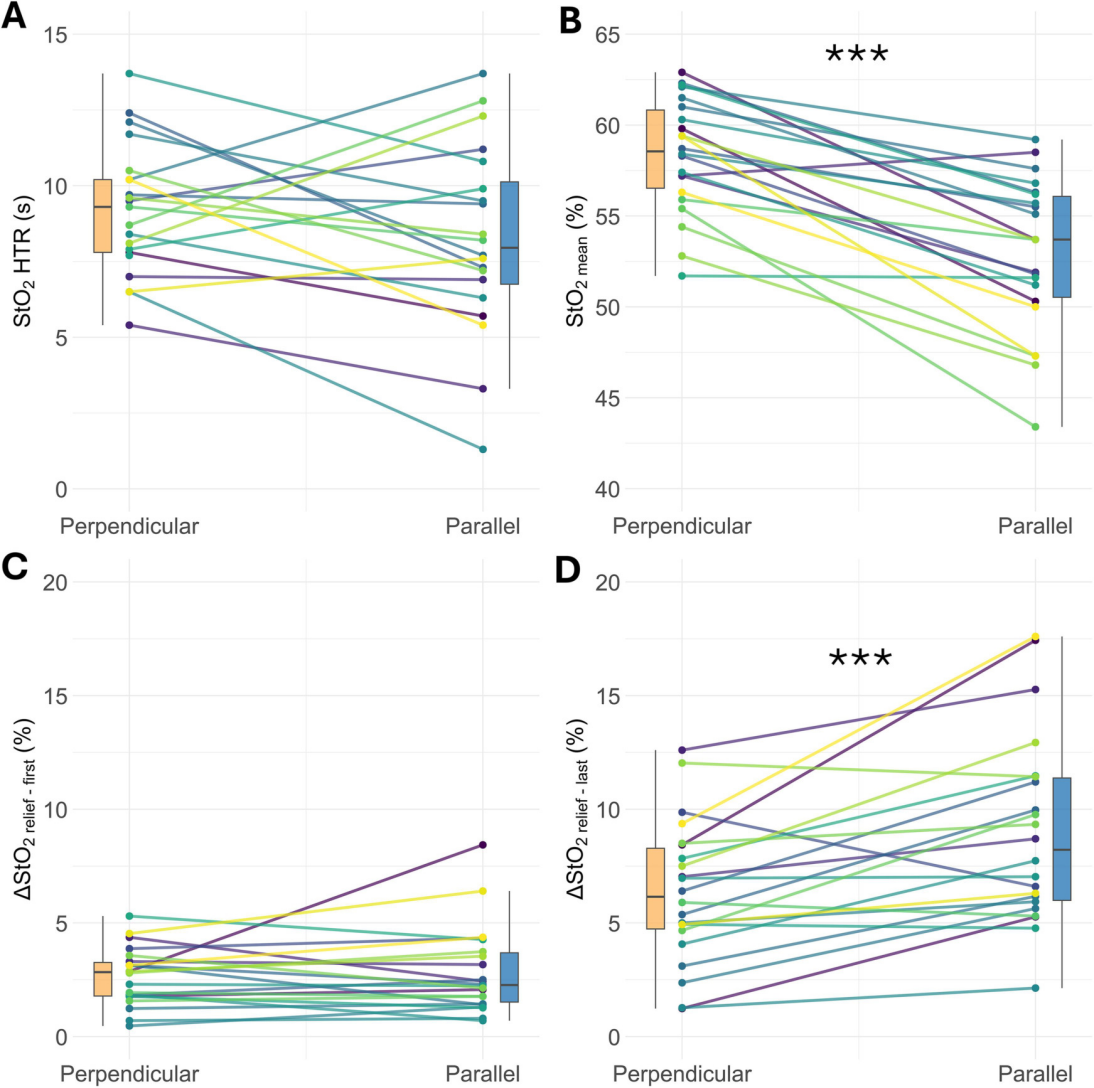
Individual responses and boxplots for perpendicular and parallel optode orientations. Boxplots show the median, interquartile range (IQR), and minimum and maximum values not greater than 1.5*IQR. **(A)** StO₂ HTR: StO₂ half-time recovery after cuff release. **(B)** StO₂ _mean_: mean muscle saturation during the incremental test. **(C)** *Δ*StO₂ _relief—first_: reoxygenation between contractions during the first stage of the incremental test. **(D)** *Δ*StO₂ _relief—last_: reoxygenation between contractions during the last stage of the test. ***Statistically significant difference (*p* < 0.001).

During the vascular occlusion test, StO₂ HTR was similar between optode orientations [mean difference ± SD, 1.0 ± 3.0 s, *p* = 0.145, *g* = 0.33 *t*(19) = 1.52, 95% CI (−0.38, 2.41)]. During the incremental endurance test, StO₂ _mean_ was 5.5 ± 3.4% lower using a parallel optode orientation than using a perpendicular orientation [*t*(21) = 7.69, *p* < 0.01, 95% CI (4.01, 6.99), *g* = 1.56]. No significant difference was found between the two optode orientations for *Δ*StO₂ _relief—first_ (−0.2% ± 1.5, *V* = 118, *p* = 0.799, *r_rb_* = 0.06). In contrast, *Δ*StO₂ _relief—last_ was 2.7 ± 2.9% higher in the parallel orientation than in the perpendicular orientation [*t*(21) = −4.26, *p* < 0.001, 95% CI (−3.97, −1.36), *g* = −0.87]. Individual responses are shown in Figure 4.

## 4 Discussion

This study aimed to characterize forearm morphology at different sites along the forearm and investigate the effects of NIRS optode orientation on haemodynamic outcome measures during rock-climbing relevant finger flexor tasks.

The main findings were as follows: (1) only a fraction of the FDP is located within 20 mm from the surface, (2) the reference site effectively avoids the FCR and FCU, and (3) the orientation of the NIRS optodes did not significantly influence StO₂ HTR or StO₂ recovery between contractions at low intensities. However, at moderate intensities, parallel optode placement resulted in higher StO₂ recovery and lower mean StO₂ than perpendicular placement, indicating an intensity-dependent effect of optode orientation on the haemodynamic outcomes.

### 4.1 Forearm morphology

When the forearm is pronated, adipose tissue and FDS accounted for an average of 93.5% of the tissue within a depth of 20 mm at the reference site. This depth is particularly relevant as most NIRS devices used to investigate the forearm musculature have an inter-optode distance of up to 40 mm, which is assumed to sample tissue to a depth of approximately 20 mm (2, 4, 10, 15, 18). Consequently, the NIR signal is likely to predominantly reflect haemodynamic changes in the FDS, with the FDP contributing to a much lesser degree. However, the precise contribution of each tissue cannot be determined for an individual measurement, let alone for a group of individuals, because the NIR signal inherently captures the entire flexor region. Therefore, we propose the use of “flexores digitorum” as a more accurate term to describe the investigated musculature in NIRS-based studies.

Therefore, care must be taken when interpreting studies aimed at investigating FDP oxygenation during intermittent finger flexor tests or climbing. For example, Feldman et al. (26) investigated the effects of finger-hang tests with varying intensities on haemodynamic measures in the FDP and their correlation with performance. They used inter-optode distances of 12.5 and 25 mm, which means that a penetration depth of 12.5 mm can be expected. Given the anatomical depth of the FDP, it is likely that this muscle contributed minimally, if at all, to the recorded NIRS signal. This limitation may partly explain the absence of observed associations between haemodynamic measures and performance, where others have reported (1).

Distinguishing between the FDP and FDS is important because muscle activity varies depending on the grip position and hold depth (27). Small holds resulted in higher FDP activity, regardless of the grip type used. When gripping holds the size of the distal phalanx or larger, the FDS experiences increased tension, particularly in a crimp position (28, 29). Therefore, the grip position and hold size used during intermittent endurance tests may influence NIRS-derived haemodynamic measurements, highlighting the importance of standardisation and accurate reporting. Moreover, future research should investigate NIRS probe placement, specifically focusing on the FDS.

The study sample mainly consisted of men and lacked the power to analyse sex differences separately; therefore, the results should be interpreted with caution. Nonetheless, the present study found that the tissue thicknesses of FDS and FDP were much greater in men compared to women. These results are consistent with those of a previous study showing significant differences between sexes in the extensor digitorum communis thickness (30). Future research should prioritize more balanced recruitment by sampling more women to enable a more comprehensive evaluation of sex differences in muscle thickness.

We hypothesize that forearm circumference could serve as a simple field measure for estimating muscle thickness in climbers. Although it showed only a weak association with FDS thickness, unaffected after adjusting for finger flexor MVC or sex, forearm circumference was strongly correlated with FDP thickness. Our findings suggest that the circumference measured at the reference site is largely determined by the FDP muscle belly and may, therefore, be suitable for tracking climbing-specific muscle hypertrophy.

Among the three measurement sites evaluated, the reference site was the most effective in minimizing interference from adjacent muscles. Nevertheless, the muscle belly of the PL was identifiable and overlaid the FDS in 32% of the participants. Owing to its anatomical location, it is not possible to avoid PL in individuals who present with it. However, its influence on the FDS thickness appeared minimal, and it did not fully overlap with the FDS in any case. Owing to the relatively low prevalence of PL in the current sample, it was not possible to assess its potential effects on haemodynamic outcomes; however, any impact is expected to be limited, given the small size of this muscle.

### 4.2 Optodes positioning

Optode orientation should be considered alongside accurate site selection. Previously, the effects of muscle fibre orientation relative to the NIRS optode have been studied in different bovine muscle samples (19) and in the human biceps brachii (20). These studies demonstrated clear differences in light propagation when optodes were positioned perpendicular rather than parallel to muscle fibres. The present study extends these findings by comparing perpendicular and parallel optode placement in the finger flexors during exercise, rather than at rest. Parallel optode placement resulted in greater *Δ*StO₂ ranges at moderate exercise intensity and lower mean StO₂ during exercise. These results support previous findings and suggest that the anisotropic properties of muscle tissue influence NIRS-derived outcome measures during exercise.

The reference proved to be most effective in avoiding FCR and FCU compared to the other sites. However, the forearm muscles lie in very close proximity to each other, and a perpendicular optode placement could increase the risk of (partially) capturing unwanted muscles. Furthermore, as mentioned above, parallel placement resulted in larger *Δ*StO₂ ranges, particularly at moderate exercise intensity, enhancing its sensitivity to detect StO₂ changes. Collectively, these findings support maintaining the current reference site to place the NIRS optodes, while ensuring that they are placed parallel to the underlying muscle fibres.

Ultrasound imaging proved to be a reliable method for assessing ATT and FDS thickness and enables the identification of adjacent anatomical structures. As such, it offers clear potential for optimizing NIRS optode placement on an individual basis by allowing practitioners to select locations that minimize interference from neighbouring muscles, arteries, and veins. However, despite these advantages, ultrasound is not universally accessible to researchers and practitioners, and its routine use prior to each NIRS assessment is impractical in most applied settings. Considering these constraints, the recommendations proposed in this study provide a more feasible approach while maintaining methodological rigour and consistency.

#### 4.3 Strengths and limitations

Several limitations of the current study must be acknowledged. First, muscle thickness was assessed only during rest. As muscle morphology changes during contraction, the results may differ during exercise.

Second, the incremental endurance test was submaximal, limiting the interpretation of the results to low and moderate intensities. Future research should examine whether optode orientation also influences haemodynamic outcomes at higher exercise intensities.

Third, the test–retest reliability of the haemodynamic outcome variables was not assessed in the current study, and the reliability of the perpendicular optode orientation has not been previously established. Consequently, it cannot be excluded that the observed differences may partly reflect the measurement variability.

The present study also has several strengths. To the best of our knowledge, this is the first study to investigate forearm morphology in climbers using US imaging. In addition, the test–retest reliability measures of the US measurements indicated that the findings were reproducible, supporting the use of this technique as a reliable method for assessing forearm tissue thickness when performed by a trained physiotherapist. Furthermore, a 15 min washout period was implemented in Part 2 of the study to minimize potential carry-over effects between the two optode orientations. Subsequent *post hoc* analyses revealed no order effects for any of the haemodynamic outcomes, suggesting that the duration of the washout period was sufficient to prevent residual influences between conditions.

### 4.4 Practical applications

In conclusion, this study demonstrated that the selected reference site, defined as 33% of the distance from the medial epicondyle of the humerus to the lunate bone, is appropriate for assessing finger flexors using NIRS. However, caution is required when attributing the signal specifically to the FDS or FDP, as it is more likely to reflect the broader flexor digitorum region. Therefore, we recommend the term flexores digitorum as a more accurate descriptor. Shifting the optodes proximally or distally may increase the likelihood of assessing adjacent muscles such as the FCR and FCU. We also recommend standardising optode placement parallel to the underlying muscle fibres, as this orientation appears to be more sensitive to changes in muscle oxygenation during climbing-specific finger contractions.

## Data Availability

The raw data supporting the conclusions of this article will be made available by the authors, without undue reservation.
