# Supplementary material for: Methodological considerations for near-infrared spectroscopy assessments in rock climbers: impact of forearm morphology and optode placement
Source: Front Sports Act Living. 2026 Jun 3;8:1845130. doi: 10.3389/fspor.2026.1845130 (PMC13272033; doi:10.3389/fspor.2026.1845130)
Supplement: Supplementary file 1 [file Datasheet1.pdf]

Table S1. Tissue thicknesses grouped by sex

	Female (n = 9)	Male (n = 19)
Proximal		
ATT	3.1 ± 0.9	2.8 ± 1.0
FDS	13.6 ± 3.7	16.1 ± 4.0
FDP	14.1 ± 3.7	20.6 ± 3.7
ATT + FDS	16.7 ± 3.8	18.9 ± 4.1
ATT + FDS + FDP	30.8 ± 5.3	39.5 ± 5.6
Reference - Supinated		
ATT	3.2 ± 1.0	2.7 ± 0.6
FDS	13.0 ± 3.4	15.7 ± 3.0
FDP	15.9 ± 3.9	22.6 ± 3.8
ATT + FDS	16.1 ± 3.5	18.3 ± 3.1
ATT + FDS + FDP	32.0 ± 5.3	40.9 ± 4.9
Distal		
ATT	3.1 ± 0.9	2.6 ± 0.6
FDS	11.6 ± 2.5	14.7 ± 2.7
FDP	16.5 ± 3.4	23.1 ± 2.9
ATT + FDS	14.6 ± 2.6	17.3 ± 2.8
ATT + FDS + FDP	31.1 ± 4.3	40.4 ± 4.1
Reference - Pronated		
ATT	3.1 ± 0.8	2.8 ± 0.8
FDS	13.2 ± 2.6	17.1 ± 3.7
FDP	18.8 ± 4.7	23.1 ± 4.2
ATT + FDS	16.4 ± 2.7	19.9 ± 3.8
ATT + FDS + FDP	35.2 ± 5.4	43.0 ± 5.6

Notes: ATT: Adipose tissue thickness, FDS: Flexor digitorum superficialis, FDP: flexor digitorum profundus. Mean ± SD are presented.
